# Supplementary material for: Chromothripsis during telomere crisis is independent of NHEJ, and consistent with a replicative origin
Source: Genome Res. 2019 May;29(5):737–49. doi: 10.1101/gr.240705.118 (PMC6499312; doi:10.1101/gr.240705.118)
Supplement: Supplemental Material [file supp_gr.240705.118_Supplemental_file_1.zip › contigs/annotated_contigs/DB51/contig.2.DB51_length_484_mean_cov_3.85123966942.docx]

**DB51_length_484_mean_cov_3.85123966942**

```
TAAACTACAGTGACATTCTGTAGGCTTCGAATTTCTGACACAATCACAACTAATATAATTGGTAGTACAATATGTTGGCTTTTCTATAT
 >chr20:20177573-20177832 + E=2e-141
TGACAAAATAGAGACTGAAAACTGTGCAGAAATGCTCTAAGAGCATGAAGATCTAATAATTTCTTATGATACGTGAGTACAATGCCAAC

ATGATCCTATTCTGTTCCTTCAAGCCAAGAACATTTCAGTGATGAATCAATAGAATAAATTAATTCTATTTTAGAAGA|CTG|AATTAC
                                                                               >chr6:74
TTCCCTTGGCTTTAGACAAGGGATGCACTGACTTTCCTCAATCACAGGACAGAATAAACAGTCTCAAAAACACAGTTTTCTCCCTTCCT
769802-74770032 + E=8e-121
GATGCCTAACTGTCAAGCTGACCTCCATTACCTCTTAGTCTTCTTATTTTTTTGGTGGGTATGTGTGGGAAGTAGGGATTCATTGTAAA

GCAAGACTTCTGGATTATTAGGTTGGTACACAAGTAATTGT
```
